
JOURNAL INFORMATION
==============================
NLM Title Abbreviation: CPT Pharmacometrics Syst Pharmacol
Journal ID: CPT Pharmacometrics Syst Pharmacol
NLM Journal ID: 101580011
Journal ID: PSP4

CPT: Pharmacometrics & Systems Pharmacology

EISSN: 2163-8306
Publisher: Wiley

ARTICLE INFORMATION
==============================
PMCID: PMC11474838
DOI: 10.1002/psp4.12989
Article ID: PSP412989
Article version: 1
Subjects: Issue Information, Issue Information

Issue Information

Electronic publication date: 2024 Jun 15
Publication date: 2024 Jun
Volume: 13
Issue: 6
First page: 905
Last page: 908
Copyright: © 2024 ASCPT
License: This is an open access article under the terms of the http://creativecommons.org/licenses/by/4.0/ License, which permits use, distribution and reproduction in any medium, provided the original work is properly cited.
License URL: https://creativecommons.org/licenses/by/4.0/

Figure count: 0
Table count: 0
Page count: 3

source-schema-version-number: 2.0
cover-date: June 2024
details-of-publishers-convertor: Converter:WILEY_ML3GV2_TO_JATSPMC version:6.4.9 mode:remove_FC converted:15.10.2024

==============================

An Official Journal of ASCPT and ISoP

### CPT: Pharmacometrics & Systems Pharmacology

Open Access

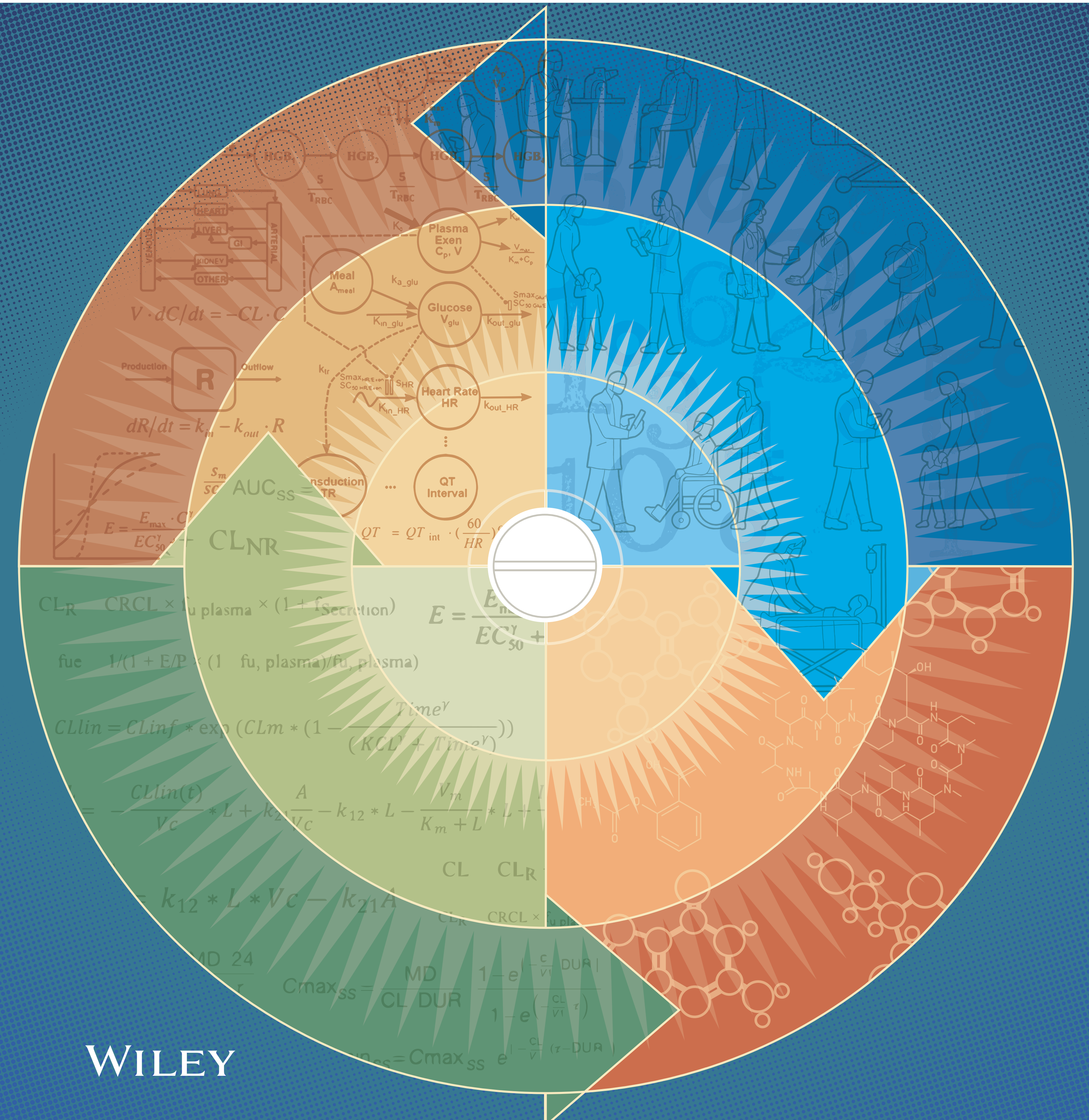

WILEY

### CPT: Pharmacometrics & Systems Pharmacology

WILEY

### Editor-in-Chief

France Mentré, MD, PhD, *School of Medicine of University Paris Diderot, Paris, France*

### Deputy Editor-in-Chief

Karen Rowland Yeo, PhD, *Certara UK Ltd., Sheffield, UK*

### Associate Editors

Paolo Denti, PhD, *University of Cape Town, Cape Town, South Africa*
Rajat Desikan, PhD, *GSK, Stevenage, UK*
Jonathan French, ScD, *Johnson & Johnson Innovative Medicine, Spring House, PA, USA*
Stefanie Hennig, MSc, PhD, *Certara Strategic Consulting, Brisbane, Australia*
Priya Jayachandran, PharmD, *Regeneron Pharmaceuticals, Inc., San Francisco, CA, USA*
Andrew Hooker, PhD, *Uppsala University, Uppsala, Sweden*
Sriram Krishnaswami, PhD, *Pfizer, Groton, CT, USA*
Ping Zhao, PhD, *Bill & Melinda Gates Foundation, Seattle, WA, USA*

### Editors-in-Training

Brian Cicali, PhD, *University of Florida, College of Pharmacy, Orlando, FL, USA*
Jane Knöchel, PhD, *AstraZeneca , Mölndal, Sweden*

### American Society for Clinical Pharmacology and Therapeutics

***Chief Executive Officer***
Abigail W. Gorman, *Alexandria, VA*
***Chief Operating Officer***
Elise S. Laffman-Johnson, *Alexandria, VA*
***Managing Editor***
Alethea B. Gerding, *Alexandria, VA*
***Deputy Managing Editor***
Alaina Webster, *Alexandria, VA*
***Senior Editorial Coordinator***
Aimee Greene, *Alexandria, VA*

### Editorial Board

Vivaswath S. Ayyar, PhD, *Janssen Research & Development, Spring House, PA*
Robert Bies, PharmD, PhD, *State University of New York at Buffalo, Buffalo, NY, USA*
Marc Birtwistle, PhD, *Clemson University Clemson, SC, USA*
Yuan Chen, PhD, *Genentech South San Francisco, CA, USA*
Marylore Chenel, PharmD, PhD, *Pharmetheus Uppsala, Sweden*
Emmanuel Chigutsa, PhD, *Eli Lilly and Company, Indianapolis, IN, USA*
Teresa Dalla Costa, PhD, *Federal University of Rio Grande do Sul - UFRGS Porto Alegre, Brazil*
Narendra M Dixit, PhD, *Indian Institute of Science, Bengaluru, India*
Anne-Gaelle Dosne, PharmD, PhD, *Janssen Research & Development, Beerse, Belgium*
Thomas Dumortier, Eng, MSc, *Novartis Pharma AG, Basel, Switzerland*
James M. Gallo, PharmD, PhD, *University at Buffalo, Buffalo, NY, USA*
Wei Gao, PhD, *EMD Serono, Billerica, MA, USA*
Jérémie Guedj, PhD, *INSERM, Paris, France*
Manish Gupta, PhD, FCP, *GenMab; Princeton, NJ, USA*
Bart Hendriks, PhD, *Novartis Institutes for BioMedical Research Cambridge, MA, USA*
Nick Holford, MBChB, *The University of Auckland Auckland, New Zealand*
Brett A. Howell, PhD, *DILIsym Services Division, Simulations Plus, Inc., Research Triangle Park, NC, USA*
Manuel Ibarra, PhD, *Universidad de la República, Montevideo, Uruguay*
Siv Jönsson, PhD, *Uppsala University Sweden, Uppsala, Sweden*
Kristin Karlsson, PhD, *Uppsala University Uppsala, Sweden*
Helen Kastrissios, PhD, *QuanTx Consulting, Brisbane, Australia*
Ron Keizer, PhD, *InsightRX San Francisco, CA, USA*
Steven E. Kern, PhD, *Global Health Labs, Bellevue, WA, USA*
Akash Khandelwal PhD, *UCB Biosciences GmbH, Monheim am Rhein, Germany*
Andrzej Kierzek, PhD, *Certara QSP; Sheffield, United Kingdom*
Qi Liu, PhD, *US Food and Drug Administration, Silver Spring, MD*
James Lu, PhD, *Genentech, Inc., South San Francisco, CA, USA*
Rajanikanth (Raj) Madabushi, PhD, *US Food and Drug Administration, Silver Spring, MD*
Efthymios Manolis, MSc, *European Medicines Agency London, United Kingdom*
Samer Mouksassi, PharmD, PhD, *Certara, Cairo, Egypt*
Jackson K. Mukonzo, PhD, *Makerere University, Kampala Uganda*
Joakim Nyberg, PhD, *Pharmetheus, Uppsala, Sweden*
Elsje Pienaar, PhD, *Weldon School of Biomedical Engineering, Purdue University, West Lafayette, IN, USA*
Daniel Scholes Rosenbloom, PhD, *Merck & Co., Rahway, NJ, USA*
Joe Standing, PhD, *University College London London, United Kingdom*
Natasha Strydom, PhD, *Amgen, Inc., San Francisco, CA, USA*
Yuichi Sugiyama, PhD, *Riken Innovation Center Sugiyama Laboratory Yokohama, Japan*
Elin Svensson, PhD, *Uppsala University, Uppsala, Sweden*
Joel Tarning, PhD, *Mahidol-Oxford Tropical Medicine Research Unit Bangkok, Thailand*
Nadia Terranova, PhD, *Merck KGaA, Lausanne, Switzerland*
Michael Tortorici, PharmD, PhD, *Aro Biotherapeutics, Philadelphia, PA, USA*
Coen van Hasselt, PhD, *Leiden University, Leiden, the Netherlands*
Paolo Vicini, PhD, MBA, *Confo Therapeutics, Ghent, Belgium*
Camille Vong, PhD, *Novartis AG Pharma, Basel, Switzerland*
Xinyuan (Susie) Zhang, PhD, *Daiichi Sankyo, Inc. Basking Ridge, NJ, USA*

### Overview

*CPT: Pharmacometrics & Systems Pharmacology* (PSP) is an official open access journal of the American Society for Clinical Pharmacology and Therapeutics (ASCPT) and International Society of Pharmacometrics (ISoP).

*CPT: Pharmacometrics & Systems Pharmacology* is a cross-disciplinary journal devoted to publishing advances in quantitative (e.g., modeling and simulation) methods as applied in pharmacology, physiology and therapeutics in humans. The journal welcomes original research articles, reviews and tutorials that bridge the following areas: pharmacometrics, modeling and simulation as applied to the design and evaluation of clinical trials, systems pharmacology modeling, particularly with a mechanistic link to human (patho)physiology, disease modeling, “population” or mixed-effects pharmacokinetics and pharmacodynamics (PKPD) modeling, modeling and simulation to support translational research, physiologically-based pharmacokinetics (PBPK), model-based meta-analyses of clinical trials, mechanism-based pharmacokinetic-pharmacodynamic modeling, computational pharmacology, bioinformatics, comparative efficacy, effectiveness and cost-effectiveness. Systems pharmacology may involve the application of systems biology approaches to study drug activities, targets and effects. The discipline is often defined with reference to engineering and pharmacological principles as the quantitative analysis of the dynamic interactions between drugs and a biologic system that aims to understand the behavior of the system as a whole. The common focus will be on quantitative methods that improve our understanding of pharmacology and therapeutics in humans.

**Abstracting and Indexing**
- Embase (Elsevier)
- SCOPUS (Elsevier)
- MEDLINE/PubMed indexing

**Society Information**
The American Society for Clinical Pharmacology and Therapeutics (ASCPT) was founded in 1900, and consists of over 2,200 professionals whose primary interest is to advance the science and practice of clinical pharmacology and translational medicine for the therapeutic benefit of patients and society. ASCPT is the largest scientific and professional organization serving the disciplines of Clinical Pharmacology and Translational Medicine.

**All articles published in this journal are freely available to read, download, and share. Articles are published under the terms of the Creative Commons Attribution Non-Commercial License which permits use, distribution, and reproduction in any medium, provided the original work is properly cited and is not used for commercial purposes.**

**Disclaimer**
The Publisher, the American Society for Clinical Pharmacology and Therapeutics, the International Society of Pharmacometrics, and Editors cannot be held responsible for any errors in or any consequences arising from the use of information contained in this journal. The views and opinions expressed do not necessarily reflect those of the Publisher, the American Society for Clinical Pharmacology and Therapeutics, the International Society of Pharmacometrics or Editors, neither does the publication of advertisements constitute any endorsement by the Publisher, the American Society for Clinical Pharmacology and Therapeutics, the International Society of Pharmacometrics, Editors, or Authors of the products advertised.

**Publisher**
*CPT: Pharmacometrics & Systems Pharmacology* is published by Wiley Periodicals, LLC, 101 Station Landing, Suite 300, Medford, MA 02155; Telephone: 781-388-8200; Fax: 781-388-8210.

CONTENTS

CPT: Pharmacometrics & Systems Pharmacology

**PERSPECTIVE**

**EDITORIAL**

909 Moving the needle for oncology dose optimization: A call for action
**Karthik Venkatakrishnan, Priya Jayachandran, Shirley K. Seo, Piet H. van der Graaf, John A. Wagner and Neeraj Gupta**

**RESEARCH**

**CASE REPORT**

919 Comparison of dose selection based on target engagement versus inhibition of receptor–ligand interaction for checkpoint inhibitors
**Sarah A. Head, Carter Johnson, Saheli Sarkar, Andrew Matteson, Diana H. Marcantonio, Fei Hua, John M. Burke, Joshua F. Apgar and David Flowers**

**ARTICLES**

926 Physiologically based pharmacokinetic modeling of imatinib and *N*-desmethyl imatinib for drug–drug interaction predictions
**Helena Leonie Hanae Loer, Christina Kovar, Simeon Rüdesheim, Fatima Zahra Marok, Laura Maria Fuhr, Dominik Selzer, Matthias Schwab and Thorsten Lehr**

941 Joint modeling of tumor dynamics and progression-free survival in advanced breast cancer: Leveraging data from amcenestrant early phase I–II trials
**Marc Cerou, Hoai-Thu Thai, Laure Deyme, Sophie Fliscounakis-Huynh, Emmanuelle Comets, Patrick Cohen, Sylvaine Cartot-Cotton and Christine Veyrat-Follet**

954 Population pharmacokinetic-pharmacodynamic modeling of serum biomarkers as predictors of tumor dynamics following lenvatinib treatment in patients with radioiodine-refractory differentiated thyroid cancer (RR-DTC)
**Oneeb Majid, Seiichi Hayato, Sree Harsha Sreerama Reddy, Bojan Lalovic, Taro Hihara, Taisuke Hoshi, Yasuhiro Funahashi, Jagadeesh Aluri, Osamu Takenaka, Sanae Yasuda and Ziad Hussein**

970 A population pharmacokinetic model using allometric scaling for baricitinib in patients with juvenile idiopathic arthritis
**Rodney L. Decker, C. Steven Ernest II, David B. Radtke, Rona Wang, Joana Araújo, Stuart Y. Keller and Xin Zhang**

982 Clinical pharmacokinetics of leriglitazone and a translational approach using PBPK modeling to guide the selection of the starting dose in children
**Estefania Traver, Laura Rodríguez-Pascau, Uwe Meya, Guillem Pina, Silvia Pascual, Sonia Poli, David Eckland, Jeroen van de Wetering, Alice Ke, Andreas Lindauer, Marc Martinell and Pilar Pizcueta**

994 Quantitative evaluation of trastuzumab deruxtecan pharmacokinetics and pharmacodynamics in mouse models of varying degrees of HER2 expression
**Christina Vasalou, Theresa A. Proia, Laura Kazlauskas, Anna Przybyla, Matthew Sung, Srinivas Mamidi, Kim Maratea, Matthew Griffin, Rebecca Sargeant, Jelena Urosevic, Anton I. Rosenbaum, Jiaqi Yuan, Krishna C. Aluri, Diane Ramsden, Niresh Hariparsad, Rhys D. O. Jones and Jerome T. Mettetal**

1006 Population pharmacokinetics of intraperitoneal irinotecan and SN-38 in patients with peritoneal metastases from colorectal origin
**Pascale C. S. Rietveld, Sebastiaan D. T. Sassen, Niels A. D. Guchelaar, Ruben A. G. van Eerden, Nadine L. de Boer, Teun B. M. van den Heuvel, Jacobus W. A. Burger, Ron H. J. Mathijssen, Birgit C. P. Koch and Stijn L. W. Koolen**

1017 Tumor growth and overall survival modeling to support decision making in phase Ib/II trials: A comparison of the joint and two-stage approaches
**Mathilde Marchand, Antonio Gonçalves, François Mercier, Pascal Chanu, Jin Y. Jin, Jérémie Guedj and René Bruno**

1029 Physiologically based pharmacokinetics modeling and transporter proteomics to predict systemic and local liver and muscle disposition of statins
**Luna Prieto Garcia, Anna Vildhede, Pär Nordell, Christine Ahlström, Ahmed B. Montaser, Tetsuya Terasaki, Hans Lennernäs and Erik Sjögren**

1044 Concentration-QTc and cardiac safety analysis of single and multiple zavegepant nasal spray doses in healthy participants to support approval
**Jim H. Hughes, Richard Bertz, Rajinder Bhardwaj, Mary K. Donohue, Jennifer Madonia, Matt S. Anderson, Beth A. Morris, Robert S. Croop and Jing Liu**

1055 Population pharmacokinetics and exposure–response analyses of polatuzumab vedotin in patients with previously untreated DLBCL from the POLARIX study
**Rong Deng, Leonid Gibiansky, Tong Lu, Christopher R. Flowers, Laurie H. Sehn, Qi Liu, Priya Agarwal, Michael Z. Liao, Randall Dere, Calvin Lee, Gabriel Man, Jamie Hirata, Chunze Li and Dale Miles**

1067 Translational kinetic-pharmacodynamics of mRNA-6231, an investigational mRNA therapeutic encoding mutein interleukin-2
**Ivana Liric Rajlic, Beatriz Guglieri-Lopez, Nabeel Rangoonwala, Vijay Ivaturi, Linh Van, Simone Mori, Brian Wipke, Douglas Burdette and Husain Attarwala**
